# A cross-sectional assessment of health-related quality of life in Chinese patients with chronic hepatitis c virus infection with EQ-5D

**DOI:** 10.1186/s12955-018-0941-8

**Published:** 2018-06-15

**Authors:** Rui Huang, Huiying Rao, Jia Shang, Hong Chen, Jun Li, Qing Xie, Zhiliang Gao, Lei Wang, Jia Wei, Jianning Jiang, Jian Sun, Jiaji Jiang, Lai Wei

**Affiliations:** 1 Peking University People’s Hospital, Peking University Hepatology Institute, Beijing Key Laboratory for Hepatitis C and Immunotherapy for Liver Disease, Beijing, China; 2 Henan Provincial People’s Hospital, Zhengzhou, China; 3 First Hospital of Lanzhou University, Lanzhou, China; 4 First Affiliated Hospital with Nanjing Medical University, Nanjing, China; 5 Shanghai Ruijin Hospital, Shanghai, China; 6 Third Affiliated Hospital of Sun Yat-sen University, Guangzhou, China; 7 Second Hospital of Shandong University, Jinan, China; 8 First Affiliated Hospital of Kunming Medical College, Kunming, China; 9 First Affiliated Hospital of Guangxi Medical University, Nanning, China; 10 Nanfang Hospital, Southern Medical University, Guangzhou, China; 11 First Affiliated Hospital of Fujian Medical University, Fuzhou, China

**Keywords:** Health related quality of life, Chronic hepatitis C, Euroqol EQ-5D, China

## Abstract

**Background:**

Hepatitis C virus (HCV) infection is one of the most common liver infections, with a decrement in HRQoL of HCV patients. This study aims to assess Health-related quality of life (HRQoL) in Chinese patients with chronic HCV infection, and to identify significant predictors of the HRQoL in these patients of China.

**Methods:**

In this cross-sectional observational study, treatment-naïve Han ethnic adults with chronic HCV infection were enrolled. Adopting European Quality of Life scale (EQ-5D) and EuroQOL visual analogue scale (EQ-VAS) were used to qualify HRQoL. Results were reported in descriptive analyses to describe sociodemographic and clinical characteristics. Multiple linear regression analysis was applied to investigate the associations of these variables with HRQoL. Binary logistic regression analysis was performed to identify associations of these variables with HRQoL by dimensions of EQ-5D.

**Results:**

Nine hundred ninety-seven patients were enrolled in the study [median age 46.0 (37.0, 56.0) years; male 54.8%]. Mean EQ-5D index and EQ-VAS score were 0.780 ± 0.083 and 77.2 ± 14.8. Multiple Linear regression analysis showed that income (< 2000 RMB, β = − 0.134; 2000–4999 RMB, β = − 0.085), moderate or severe symptoms of discomfort (more than one symptoms, β = − 0.090), disease profile (cirrhosis, β = − 0.114), hyperlipidemia (β = − 0.065) and depression (β = − 0.065) were independently associated with EQ-5D index. Residence (the west, β = 0.087), income (< 2000 RMB, β = − 0.129; 2000–4999 RMB, β = − 0.052), moderate or severe symptoms of discomfort (more than one symptoms, β = − 0.091), disease profile and depression (β = − 0.316) were the influencing factors on EQ-VAS. Binary logistic regression indicated that disease profile and clinical depression were the major influencing factors on all five dimensions of EQ-5D.

**Conclusions:**

In this cross-sectional assessment of HCV patients in China, we indicated HRQoL of Chinese HCV patients. Significant negative associations between HRQoL and sociodemographic and clinical factors such as moderate or severe symptoms of discomfort, disease profile and depression emerged. We have to focus on optimally managing care of HCV patients and improving their HRQoL.

**Trial registration:**

ClinicalTrials.gov identifier NCT01293279. Date of registration: February 10, 2011.

## Background

Hepatitis C virus (HCV) infection is one of the most common liver infections in the world. Total global anti-HCV prevalence is estimated at 2.5% (177.5 million of HCV infected adults), ranging from 2.9% in Africa and 1.3% in Americas [1]. With chronic, life-long infections, hepatitis C virus causes liver cirrhosis and hepatocellular carcinoma (HCC) progressively. Globally, viral hepatitis accounted for 1.45 million deaths in 2013, half of which were caused by hepatitis C infections [2]. Chronic hepatitis C infection is a public health issue of utmost importance in the world, especially in China, which has 10 million patients suffering from this disease [3].

As the move from a disease-centered to a biopsychosocial model of medicine, health-related quality of life (HRQoL) is getting more and more concerns. It’s especially important in management of chronic diseases, including chronic HCV infections. With growing interest in HRQoL these years, there have been many studies focusing on HRQoL of patients with chronic HCV infections. Compared to the general population, patients with HCV infection were reported to have a decrement in HRQoL [4, 5]. It has been shown that the impairment in HRQoL for individuals with HCV was equivalent to, or even more severe than the impact on physical and general health experienced by patients with other common chronic conditions such as hypertension, diabetes or arthritis [6].

As known, the impact of disease on HRQoL may vary from population to population because of differences in socioeconomic status, cultural heritage, religion and life style. However, few studies have evaluated HRQoL of patients with HCV in China [7]. EQ-5D is a standardised instrument for use as a measure of health outcome (http://www.euroqol.org/about-eq-5d.html), widely used in measurement of HRQoL in certain patients, and also in those with HCV infection [8, 9]. Basing on the widely use of EQ-5D worldwide, we applied it in our study. The aim of this study was to assess HRQoL in Chinese patients with chronic HCV infection, to investigate whether variables related to sociodemographic characteristics, degree of liver impairment and clinical comorbidities are independently associated with HRQOL, and finally, to identify significant predictors of the HRQoL in these patients of China.

## Methods

This study aims to assess HRQoL in Chinese patients with chronic HCV infection, and to identify significant predictors of the HRQoL in these patients of China.

### Study population

This study was one part of the cross-sectional observational study, CCgenos (ClinicalTrials.gov identifier NCT01293279) [10]. To investigate the distribution of viral and host genotypes, only Han ethnic HCV treatment-naïve patients were enrolled. Those patients at least 18 years of age were seen at the outpatient department of 28 university-affiliated hospitals across China between February and June 2011. HCV infection was confirmed (anti-HCV antibody and HCV RNA positive) within 90 days prior to enrollment. Patients who had received antiviral or interferon-based therapy for hepatitis C or hepatitis B were excluded; no other exclusion criteria were applied. Social-demographic and clinical information were collected within 9 days after enrollment, so was EQ-5D-3 L questionnaire. The respondents were asked to classify five dimensions of their own health status into three levels. They were asked to identify the point that they felt best illustrated their current overall health state on a visual analogue scale (VAS).

Social-demographic characteristics considered in this study included sex, age, residence (east, west, south, north, central), marital status (single, married, separated/divorced/widowed), occupation (white collar, blue collar, unemployed, other), education attainment (primary junior, junior school, high school, college graduate) and economic status (monthly family income/per person < 2000 RMB, 2000–4999 RMB, ≥5000 RMB) (1 RMB = 0.1457 USD.).

Clinical characteristics considered in this study included symptoms of discomfort (moderate or severe malaise, appetite decrease, nausea, discomfort of right hypochondrium, fever, xanthochromia, xanthurenic and other uncomfortable symptoms), disease profile (cirrhosis and chronic hepatitis, in which hepatitis without any moderate or severe uncomfortable symptoms mentioned above was considered asymptomatic, while the rest symptomatic), extrahepatic manifestations (Behcet’s disease, cryoglobulinemia, prediabetes, diabetes, hyperlipidemia, fatty liver, insulin resistance, fibromyalgia, membranoproliferative glomerulonephritis, membranous nephropathy, multiple myeloma, Raynaud’s syndrome, rheumatoid arthritis, Sjögren syndrome, systemic lupus erythematosus, thyroid dysfunction, skin lesions, hypertrophic cardiomyopathy, porphyria cutanea tarda, lichen planus, vasculitis, pulmonary fibrosis). In addition, comorbidities diagnosed by doctors including hepatitis B, fatty liver, hyperlipidemia and diabetes were considered. Beck’s depression score, which suggested clinical depression with score ≥ 17 was calculated.

Cirrhosis was diagnosed by liver biopsy or the presence of ascites, hepatic encephalopathy, upper gastrointestinal bleeding, or Child-Turcotte-Pugh score > 7, or by any two of the following criteria: radiologic imaging showing nodular liver or splenomegaly, platelet count < 100 × 10^9^/L in the absence of other explanations, liver stiffness >13kPA, or endoscopic detection of gastroesophageal varices.

### Ethical approval

The study was conducted in accordance with the International Society for Pharmacoepidemiology guidelines for Good Epidemiology Practices and applicable regulatory requirements. All patients provided written informed consent before participating.

### EQ-5D

We used an approved Chinese version of the EQ-5D-3 L which has been tested for its validity and reliability in populations in mainland China [11, 12], EQ- 5D-3 L is the simplest multidimensional measure, composed of the EQ-5D descriptive system and the EQ visual analogue scale (EQ-VAS). The EQ-5D-3 L descriptive system classified patients’ health status at three levels of severity (no, moderate, or severe problems) in five dimensions (mobility; self-care; usual activities; pain/ discomfort; anxiety/depression), resulting in scores that can be converted into a single index value for health status (1 = full health; 0 = dead). Since no verified Chinese value set is available, the index value was assigned using a Japanese time trade-off value set [13], in which intercept was 0.152; mobility (none: 0.000; moderate: 0.075; severe: 0.418), self-care (none: 0.000; moderate: 0.054; severe: 0.102), usual activity (none: 0.000; moderate:0.044; severe: 0.133); pain/discomfort (none: 0.000; moderate: 0.080; severe: 0.194) and anxiety/depression (none: 0.000; moderate: 0.063; severe: 0.112) had correspondent values in three grade. In addition to the self-classifier, the EQ-5D contains a 20 cm visual analog scale (VAS) ranging from 0 (worst imaginable health) to 100 (best imaginable health) along which the respondent rates their health today. The index-based utility scores can be used to compare a burden of disease across different conditions. A single summary score was generated by applying societal preference weights to a health state classifier completed by patients.

### Statistical analysis

Descriptive analysis of patients’ demographic information was performed. Categorical variables were measured as percentages while continuous variables were expressed as mean ± standard deviation. Mann–Whitney (gender, fatty liver, hyperlipidemia, diabetes, Beck’s depression score) and Kruskal Wallis (age, residence, marital status, occupation, education, income, symptoms of discomfort, disease profile, and extrahepatic manifestation) tests were used as Kolmogrov-Smirnov test revealed nonnormal distribution of the data. A multiple linear regression analysis was applied to investigate the associations of social-demographic and clinical characteristics with HRQoL to select those linked to EQ index and EQ-VAS. A binary logistic regression analysis was performed to identify associations of these variables with HRQoL by dimensions of EQ-5D to select those linked to impairment in individual dimension of the EQ-5D instruments. A statistical value of *p* < 0.05 was taken as significant. All analyses were performed using SPSS 23.0 (SPSS Inc., Chicago, IL).

## Results

### Study population

One thousand and twelve patients enrolled, in whom 997 patients met the protocol criteria for inclusion, while 15 were excluded for protocol violations.

### Patient sociodemographic and clinical status

The sociodemographic characteristics of the patients were listed in Table 1. Patients had a median of 46 years of age and 546 (54.8%) were males.

**Table 1:** Sociodemographic characteristics of patients. (near Line 263)

Parameter	
Gender	
Male, *n* (%)	546(54.8)
Female, *n* (%)	451(45.2)
Age, median years, (Q1, Q3)	46.0 (37.0, 56.0)
18–39, *n* (%)	324 (32.5)
40–59, *n* (%)	525 (52.7)
≥ 60, *n* (%)	148 (14.8)
Residence	
East, *n* (%)	206 (20.7)
West, *n* (%)	217 (21.8)
South, *n* (%)	152 (15.2)
North, *n* (%)	181 (18.2)
Central, *n* (%)	241 (24.2)
Marital status	
Single	116 (11.6)
Married	842 (84.5)
Separated/Divorced/Widowed	39 (3.9%)
Occupation	
White collar	260 (26.1)
Blue collar	414 (41.5%)
Unemployed	179 (18.0)
Other	144 (14.4)
Education	
Primary School, *n* (%)	177 (17.7)
Junior school, *n* (%)	271 (21.8)
High-school, *n* (%)	273 (27.4)
College graduate, *n* (%)	276 (27.6)
Monthly family income/per person^a^	
< 2000 RMB	558 (56.0)
2000–4999 RMB	352 (35.3)
≥ 5000 RMB	87 (8.7)

^a^1 RMB = 0.1457 USD

The clinical characteristics of the patients were listed in Table 2. 856 (86.1%) patients didn’t report any moderate or severe symptoms of discomfort, such as appetite decrease, nausea. Cirrhosis was reported in 101(10.1%) patients. 41 (4.1%) patients had a coinfection of hepatitis B and C. Beck’s depression score more than 17, suggesting clinical depression were seen in 168 (16.9%) patients.

**Table 2:** Clinical characteristics of patients. (near Line 273)

Parameter	
Number of moderate or severe symptoms of discomfort, *n* (%)^a^	
0	858 (86.1)
1	93 (9.3)
≥ 2	46(4.6)
Disease profile, *n* (%)	
Chronic hepatitis, asymptomatic^b^	404 (40.5)
Chronic hepatitis, symptomatic	492 (49.3)
Cirrhosis	101 (10.1)
Extrahepatic manifestations, *n* (%)^c^	
Yes	166 (16.6)
No	831 (83.4)
Hepatitis B	
Yes	41 (4.1)
No	956 (95.9)
Fatty liver	
Yes	242 (24.3)
No	755 (75.7)
Hyperlipidemia	
Yes	22 (2.2)
No	975 (97.8)
Diabetes	
Yes	81 (8.1)
No	916 (91.9)
Beck’s Depression Score, median (Q1, Q3)	9.2 (3.0, 13.0)
< 17	829 (83.1
≥ 17	168 (16.9)

^a^Uncomfortable symptoms: moderate or above malaise, appetite decrease, nausea, discomfort of right hypochondrium, fever, xanthochromia, xanthurenic and other uncomfortable symptoms. ^b^Chronic hepatitis, asymptomatic: chronic hepatitis without any discomfortable complain above. ^c^Extrahepatic manifestation: Behcet’s disease, cryoglobulinemia, prediabetes, diabetes, hyperlipidemia, fatty liver, insulin resistance, fibromyalgia, membranoproliferative glomerulonephritis, membranous nephropathy, multiple myeloma, Raynaud’s syndrome, rheumatoid arthritis, Sjögren syndrome, systemic lupus erythematosus, thyroid dysfunction, skin lesions, hypertrophic cardiomyopathy, porphyria cutanea tarda, lichen planus, vasculitis, pulmonary fibrosis

### Health-related quality of life

Mean EQ-5D descriptive score and EQ-VAS were 0.780 ± 0.083 and 77.2 ± 14.8, respectively. 79 (7.9%), 24(2.4%), and 71(7.1%) patients reported some problem (moderate or severe) in the mobility, self-care and usual activities respectively, whereas 343 (34.2%) patients indicated some pain or discomfort (moderate or severe) in the fourth domain and 341 (34.1%) reported moderate or severe anxiety or depression in the fifth domain as shown in Table 3.

**Table 3:** EQ-5D results of patients with hepatitis C. (near Line 304)

EQ-5D	None, *n* (%)	Moderate, *n* (%)	Severe, *n* (%)
Mobility difficulties	918 (92.1)	74 (7.4)	5 (0.5)
Self-care difficulties	973 (97.6)	22 (2.2)	2 (0.2)
Usual activities difficulties	926 (92.9)	68 (6.8)	3 (0.3)
Pain/discomfort	655 (65.7)	333 (33.4)	9 (0.9)
Anxiety/depression	656 (65.8)	313 (31.4)	28 (2.8)
EQ-5D index	0.780 ± 0.083		
EQ-5D VAS	0.780 ± 0.083		

### EQ-5D by sociodemographic and clinical characteristics

EQ-5D index was significantly different by gender (0.792 ± 0.087 vs 0.777 ± 0.078, *p* = 0.000), age (*p* = 0.000), marital status (*p* = 0.000), education (*p* = 0.000), income (*p* = 0.001), symptoms of discomfort (*p* = 0.000), disease profile (*p* = 0.000), fatty liver (0.772 ± 0.100 vs 0.788 ± 0.083, *p* = 0.041), hyperlipidemia (0.740 ± 0.110 vs 0.785 ± 0.087, *p* = 0.008), diabetes (0.767 ± 0.101 vs 0.786 ± 0.086, *p* = 0.037) and Beck’s depression score (0.798 ± 0.074 vs 0.712 ± 0.115, *p* = 0.000). EQ-VAS was significantly different by age (*p* = 0.000), residence (*p* = 0.000), marital status (*p* = 0.006), income (*p* = 0.001), symptoms of discomfort (*p* = 0.000), disease profile (*p* = 0.000), symptoms of discomfort (*p* = 0.000), Beck’s depression score (79.5 ± 13.1 vs 66.0 ± 17.4, *p* = 0.000). (Table 4 and Table 5).

**Table 4:** Mean EQ-5D indexes and EQ-VAS scores by sociodemographic characteristics. (near Line 317)

Parameter	Mean EQ-5D index	SD	*p* value	Mean EQ-VAS score	SD	*p* value
Gender^b^						
Males	0.792	0.087	0.000	77.7	14.7	0.222
Females	0.777	0.078		76.7	14.8	
Age^a^						
18–39	0.813	0.054	0.000	82.2	11.2	0.000
40–59	0.790	0.077		78.6	13.8	
≥ 60	0.771	0.081		75.4	14.1	
Residence^a^						
East	0.781	0.091	0.115	74.4	12.8	0.000
West	0.788	0.090		79.9	15.2	
South	0.789	0.073		76.7	15.0	
North	0.776	0.081		77.6	15.6	
Central	0.790	0.077		77.3	14.9	
Marital status^a^						
Single	0.806	0.062	0.000	80.4	13.9	0.006
Married	0.785	0.083		77.1	14.8	
Separated/Divorced/Widowed	0.730	0.114		72.0	16.2	
Occupation^a^						
White collar	0.792	0.080	0.054	78.6	14.1	0.173
Blue collar	0.780	0080		76.6	14.7	
Unemployed	0.784	0.103		77.7	15.1	
Other	0.788	0.071		76.0	14.8	
Education^a^						
Primary School	0.763	0.102	0.000	75.7	16.1	0.105
Junior school	0.782	0.100		76.5	14.9	
High-school	0.785	0.072		77.1	14.8	
College graduate	0.798	0.087		79.1	13.6	
Monthly family income/per person^a^						
< 2000 RMB	0.778	0.091	0.001	75.8	15.7	0.001
2000–4999 RMB	0.790	0.074		78.5	13.7	
≥ 5000 RMB	0.811	0.057		81.5	11.5	

^a^Kruskal Wallis Test. ^b^Mann Whitney Test

**Table 5:** Mean EQ-5D indexes and EQ-VAS scores by clinical characteristics. (near Line 319)

Parameter	EQ-5D index, mean	SD	*p* value	EQ VAS, mean	SD	*p* value
Number of moderate or severe symptoms of discomfort^a^						
0	0.792	0.081	0.000	78.5	14.0	0.000
1	0.746	0.093		71.6	15.7	
≥ 2	0.719	0.087		66.0	18.7	
Disease profile^a^						
Chronic hepatitis, asymptomatic	0.804	0.081	0.000	80.7	13.8	0.000
Chronic hepatitis, symptomatic	0.774	0.076		75.2	15.0	
Cirrhosis	0.751	0.133		73.3	13.8	
Extrahepatic manifestation^a^						
Yes	0.779	0.100	0.487	76.0	15.8	0.068
No	0.787	0.086		77.7	14.5	
Hepatitis B^b^						
Yes	0.771	0.115	0.791	72.5	16.0	0.051
No	0.785	0.086		77.4	14.7	
Fatty liver^b^						
Yes	0.772	0.100	0.041	76.4	15.3	0.552
No	0.788	0.083		77.5	14.6	
Hyperlipidemia^b^						
Yes	0.740	0.110	0.008	72.5	13.0	0.052
No	0.785	0.087		77.3	14.8	
Diabetes^b^						
Yes	0.767	0.101	0.037	74.0	15.9	0.053
No	0.786	0.086		77.5	15.9	
Beck’s Depression Score^b^						
< 17	0.798	0.074	0.000	79.5	13.1	0.000
≥ 17	0.712	0.115		66.0	17.4	

^a^Kruskal Wallis Test. ^b^Mann Whitney Test

### Multiple linear regression analyses on EQ-5D

A Multiple Linear regression model was applied to investigate the effects of sociodemographic and clinical variables on HRQoL in patients with HCV (Table 6).

**Table 6:** Multiple linear regression analyses on EQ-5D index score and EQ-VAS scores. (near Line 344)

Parameter	EQ-5D index						EQ-VAS score			
	B^a^ ± SE	β estimate^b^	*p* value	95.0% CI for B		B^a^ ± SE	β estimate^b^	*p* value	95.0% CI	
				Lower	Upper					
Intercept	0.839 ± 0.018		0.000	0.803	0.875	82.238 ± 3.112		0.000	76.132	88.344
Gender (Ref: Male)	− 0.007 ± 0.005	− 0.042	0.176	− 0.018	0.003	0.207 ± 0.920	0.007	0.822	−1.592	2.012
Age (Ref: ≥60y)										
18–29	0.016 ± 0.012	0.062	0.198	− 0.008	0.040	3.991 ± 2.101	0.091	0.058	− 0.132	8.113
30–39	0.007 ± 0.009	0.030	0.477	−0.012	0.025	0.968 ± 1.587	0.026	0.542	−2.147	4.083
40–49	0.011 ± 0.008	0.055	0.203	− 0.006	0.027	2.372 ± 1.433	0.072	0.098	− 0.440	5.184
50–59	0.007 ± 0.008	0.035	0.400	− 0.009	0.023	0.799 ± 1.411	0.023	0.571	− 1.970	3.569
Residence (Ref: Central)										
East	− 0.002 ± 0.008	− 0.011	0.768	− 0.018	0.013	− 2.047 ± 1.338	− 0.056	0.126	−4.672	0.579
West	0.002 ± 0.008	− 0.010	0.777	− 0.013	0.017	3.115 ± 1.280	0.087	0.015	0.603	5.627
South	− 0.001 ± 0.009	− 0.006	0.873	−0.018	0.015	− 1.004 ± 1.445	− 0.024	0.487	− 3.841	1.832
North	−0.005 ± 0.009	−0.021	0.575	−0.022	0.012	1.467 ± 1.438	0.038	0.308	−1.355	4.289
Marital status (Ref: Single)										
Married	−0.007 ± 0.011	−0.030	0.500	−0.029	0.014	−0.741 ± 1.833	−0.018	0.686	−4.339	2.857
Separated/Divorced/Widowed	−0.033 ± 0.017	−0.073	0.052	−0.066	− 0.000	−0.671 ± 2.856	−0.009	0.814	−6.277	4.934
Education (Ref: Primary school**)**										
Junior school	− 0.004 ± 0.009	−0.018	0.631	−0.021	0.013	2.410 ± 1.481	0.0062	0.104	−0.496	5.315
High-school	0.005 ± 0.007	0.023	0.538	−0.010	0.019	1.640 ± 1.256	0.049	0.192	−0.825	4.104
College graduate	0.002 ± 0.007	0.010	0.790	−0.012	0.016	0.749 ± 1.192	0.023	0.530	−1.590	3.087
Monthly family income/per person (≥5000 RMB)										
< 2000 RMB	−0.024 ± 0.010	−0.134	0.017	−0.043	− 0.004	−3.833 ± 1.669	−0.129	0.022	−7.107	−0.558
2000–4999 RMB	−0.016 ± 0.010	−0.085	0.110	−0.035	0.004	−1.612 ± 1.647	−0.052	0.328	−4.844	1.621
Number of moderate or severe symptoms of discomfort (Ref:0)										
1	−0.017 ± 0.009	−0.056	0.074	−0.035	0.002	−2.358 ± 1.588	−0.046	0.138	−5.474	0.758
≥ 2	−0.038 ± 0013	−0.090	0.003	−0.062	− 0.013	−6.410 ± 2.124	−0.091	0.003	−10.578	− 2.241
Disease profile (Ref: Chronic hepatitis, asymptomatic)										
Chronic hepatitis, symptomatic	−0.014 ± 0.006	−0.078	0.022	−0.025	− 0.002	−3.167 ± 1.003	−0.107	0.002	− 5.135	− 1.199
cirrhosis	−0.033 ± 0.010	−0.114	0.001	−0.052	− 0.014	− 4.834 ± 1.630	−0.099	0.003	− 8.032	−1.636
Fatty liver (Ref: No)	0.003 ± 0.009	0.011	0.710	−0.014	0.021	−0.629 ± 1.524	−0.013	0.680	−3.621	2.362
Hyperlipidemia (Ref: No)	−0.039 ± 0.018	−0.065	0.031	−0.074	− 0.003	−3.147 ± 3.046	−0.031	0.302	−9.125	2.831
Diabetes (Ref: No)	−0.011 ± 0.010	− 0.033	0.272	− 0.030	0.008	−2.616 ± 1.632	−0.048	0.109	− 5.819	0.586
Beck’s Depression Score (Ref: < 17)	−0.069 ± 0.007	−0.294	0.000	−0.083	− 0.054	−12.453 ± 1.220	−0.316	0.000	− 14.848	− 10.059

^a^Unstandardized sample regression coefficient

^b^Standardized sample regression coefficient

EQ-5D index was negatively associated with symptoms of discomfort, disease progression, hyperlipidemia (β = − 0.065) and Beck’s depression score (β = − 0.294). Compared to patients without any moderate or severe symptoms, those had one (β = − 0.056) or more (β = − 0.090) symptoms reported lower EQ-5D index. When compared to patients with asymptomatic chronic hepatitis, those with any symptoms (β = − 0.078) or cirrhosis (β = − 0.114) indicated lower EQ-5D index. Income was positively associated with EQ-5D index (< 2000 RMB, β = − 0.134; 2000–4999 RMB, β = − 0.085).

EQ-VAS was negatively associated with symptoms of discomfort, disease progression and Beck’s depression score (β = − 0.316). Patients with one (β = − 0.046) or more (β = − 0.091) symptoms had lower EQ-VAS. Patients with any symptoms (β = − 0.107) or cirrhosis (β = − 0.099) reported lower EQ-VAS. Patients from the west of China had highest EQ-VAS, compared to those from other regions (β = 0.087). Income was positively associated with EQ-VAS (< 2000 RMB, β = − 0.129; 2000–4999 RMB, β = − 0.052).

### Logistic regression analyses in each EQ-5D dimension

Further analyses comparing patients with and without any problem in each EQ-5D dimension by binary logistic regression indicated that disease profile and clinical depression were the major influencing factors on all the five dimensions (Table 7). Compared to asymptomatic chronic hepatitis, symptomatic chronic hepatitis and cirrhosis brought more problems in mobility, self-care, usual activities, pain/discomfort and anxiety/depression. Beck’s depression score ≥ 17 were also significantly associated with all five dimensions.

**Table 7:** Binary logistic regression analyses on having problem in each EQ-5D dimension. (near Line 359)

Independent variables	B	*p*	Odds ratio	95% CI	
				Lower	Upper
a. Dependent variable: mobility					
Age (Ref: 17-29y)					
30–39	1.526	0.183	4.598	0.488	43.360
40–49	1.318	0.257	3.736	0.383	36.450
50–59	1.938	0.094	6.948	0.719	67.187
≥ 60	2.819	0.015	16.759	1.733	162.099
Disease profile (Ref: Chronic hepatitis, asymptomatic)					
Chronic hepatitis, symptomatic	0.072	0.833	1.074	0.551	2.093
cirrhosis	1.201	0.002	3.323	1.539	7.174
Diabetes (Ref: No)	0.696	0.049	2.006	1.002	4.018
Beck’s Depression Score, mean (Ref: < 17)	0.881	0.004	2.414	1.326	4.396
b. Dependent variable: self-care					
Gender (Ref: Male)	−1.319	0.015	0.267	0.093	0.771
Residence (Ref: Central)					
East	0.098	0.085	1.103	0.292	4.160
West	0.265	0.688	1.303	0.357	4.752
South	−0.712	0.425	0.491	0.085	2.819
North	−2.111	0.043	0.121	0.016	0.939
Number of moderate or severe symptoms of discomfort (Ref:0)					
1	1.960	0.002	7.103	2.043	24.692
≥ 2	0.923	0.260	2.517	0.505	12.560
Disease profile (Ref: Chronic hepatitis, asymptomatic)					
Chronic hepatitis, symptomatic	−1.170	0.105	0.310	0.075	1.278
cirrhosis	1.333	0.046	3.793	1.023	14.062
Hyperlipidemia (Ref: No)	2.137	0.026	8.476	1.290	55.713
Beck’s Depression Score, mean (Ref: < 17)	1.256	0.015	3.512	1.275	9.672
c. Dependent variable: usual activities					
Number of moderate or severe symptoms of discomfort (Ref:0)					
1	0.707	0.061	2.028	0.968	4.253
≥ 2	1.159	0.008	3.186	1.355	7.495
Disease profile (Ref: Chronic hepatitis, asymptomatic)					
Chronic hepatitis, symptomatic	0.409	0.273	1.505	0.725	3.123
cirrhosis	1.596	0.000	4.932	2.138	11.374
Beck’s Depression Score, mean (Ref: < 17)	0.796	0.008	2.218	1.230	3.998
d. Dependent variable: pain/discomfort					
Gender (Ref: Male)	0.057	0.000	1.746	1.283	2.375
Age (Ref: 17-29y)					
30–39	0.616	0.088	1.851	0.913	3.753
40–49	0.621	0.082	1.860	0.925	3.742
50–59	0.735	0.047	2.086	1.008	4.317
≥ 60	1.011	0.009	2.748	1.285	5.876
Number of moderate or severe symptoms of discomfort (Ref:0)					
1	0.388	0.128	1.474	0.894	2.428
≥ 2	0.573	0.032	1.774	1.051	2.993
Disease profile (Ref: Chronic hepatitis, asymptomatic)					
Chronic hepatitis, symptomatic	0.767	0.000	2.154	1.527	3.038
cirrhosis	0.573	0.032	1.774	1.051	2.993
Hyperlipidemia (Ref: No)	1.092	0.033	2.981	1.090	8.150
Beck’s Depression Score, mean (Ref: < 17)	1.165	0.000	3.207	2.175	4.728
e. Dependent variable: anxiety/depression					
Gender (Ref: Male)	0.322	0.041	1.380	1.013	1.879
Monthly family income/per person (≥5000 RMB)					
< 2000 RMB	0.628	0.041	1.874	1.025	3.424
2000–4999 RMB	0.328	0.287	1.388	0.759	2.537
Disease profile (Ref: Chronic hepatitis, asymptomatic)					
Chronic hepatitis, symptomatic	0.570	0.001	1.769	1.263	2.476
cirrhosis	−0.310	0.302	0.733	0.407	1.322
Beck’s Depression Score, mean (Ref: < 17)	1.891	0.000	6.623	4.428	9.907

Old patients had more problems on the mobility dimension, and diabetes remained significantly associated with mobility (β = 0.696; *p* = 0.049; odds ratio OR = 2.006; 95% CI 1.002–4.018). Problems on self-care were significantly associated with males (β = − 1.319; *p* = 0.015; OR = 0.267; 95% CI 0.093–0.771), residence, symptoms of discomfort and hyperlipidemia (β = 2.137; *p* = 0.026; OR = 8.476; 95% CI 1.290–55.713): patients from the west had more problem of self-care than those from other regions (β = 0.265; *p* = 0.688; OR = 1.303; 95% CI 0.357–4.752); patients with moderate or severe symptoms of discomforts had seven times more likely to have problem in self-care (β = 1.960; *p* = 0.002; OR = 7.103; 95% CI 2.043–24.692). More problems in usual activities were significantly associated with ≥2 symptoms of discomforts (β = 1.159; *p* = 0.008; OR = 3.186; 95% CI 1.355–7.495). Females (β = 0.057; *p* = 0.000; OR = 2.748; 95% CI 2.748–2.375), the old age (β = 1.011; *p* = 0.009; OR = 1.746; 95% CI 1.285–5.876) and hyperlipidemia (β = 1.092; *p* = 0.033; OR = 2.981; 95% CI 1.090–8.150) remained significantly associated with pain/discomfort. Besides, more pain/discomfort were significantly associated with ≥2 symptoms of discomforts (β = 0.573; *p* = 0.032; OR = 1.774; 95% CI 1.051–2.993). Females (β = 0.322; *p* = 0.041; OR = 1.380; 95% CI 1.013–1.879) and low income (β = 0.628; *p* = 0.041; OR = 1.874; 95% CI 1.025–3.424) were significantly associated with anxiety/depression.

## Discussion

With 118.9 million HCV RNA positive cases worldwide [1], it is important to understand the effects of HCV infection on patients’ well-being and HRQoL, by assessing the health outcome reported by these patients. It would help to improve their care management. Poor HRQoL were demonstrated in these Chinese patients with chronic HCV infection. Worsening HRQoL was significantly associated with unstable marital status, residence, poor family income, more symptoms of moderate or severe discomfort, disease progression of hepatitis C and some comorbidities such as hyperlipidemia and depression. This is a multi-centric, large-sample assessment of HRQoL in Chinese patients with HCV. Although HRQoL of patients with HCV has been evaluated in several counties and regions, the current study from mainland China may provide important evidence for expanding the knowledge about the impact of chronic hepatitis C infection on health of people.

Although majority of HCV-infected patients are asymptomatic during its natural course, HCV itself may still decrease the HRQOL no matter how advanced disease progression they have [14]. Studies in European and American countries indicated that HCV patients had a clinically meaningful decrement in quality of life compared to controls [15, 16]. The relevant data from Asia was limited [17, 18], however, our result, that the mean EQ-5D index and EQ-VAS of patients with HCV infection in this study were 0.780 ± 0.083 and 77.2 ± 14.8 respectively brought the knowledge about the impact of HCV infection on Chinese patients’ HRQoL.

Sociodemographic characteristics such as residence in the west of China, stable marital status, high family income have been found to be favorable to better quality of life for patients with HCV infection. Although the association between gender and HRQoL was not significant in our study, women still reported more problems in dimensions of pain/discomfort (OR = 1.746; 95% CI 1.283–2.375) and anxiety/depression (OR = 1.380; 95% CI 1.013–1.879), which was in line with EQ-5D population health studies in both other countries and China [19–21] .Female patients with chronic hepatitis C tended to be fatigued and depressive [22], which might be the reason why they had worse HRQoL than males. What’s interesting we found was that patients from western China, who resided in less developed area of China, had significant higher EQ-VAS scores but more problems in self-care dimension, indicating that they considered themselves had relatively better health status, while their EQ-5D index didn’t show the same superiority. The reason may be that patients from western China hadn’t truly recognized their well-being and health status with less knowledge and education for hepatitis C than those from developed areas. We should make further efforts on this issue to tell the reason why discrepancy existed by different areas across China in the future.

Clinical characteristics such as more symptoms of moderate or severe discomfort, advanced disease progression, hyperlipidemia and Beck’s depression score were indicated to be associated with worse HRQoL. Patients with hyperlipidemia reported worse health status by EQ-5D index. As known, HCV infection induced hepatic steatosis and altered lipid metabolism [23]. Our results demonstrated the similar association: suffering from hyperlipidemia made EQ-5D Index significantly lower (β = − 0.065) by impacting on dimensions of self-care (OR = 8.476; 95% CI 1.290–55.713) and pain/discomfort (OR = 2.981; 95 %CI 1.090–8.150). Limited literatures focused on the impact of hyperlipidemia on the quality of life among patients with HCV infection, and the only two relevant studies didn’t show the association between hyperlipidemia and HRQoL in HCV population [24, 25], which we found in this study highlighted the potential correlation of hyperlipidemia and HRQoL in patients with chronic HCV infection.

Coinfection of HBV/HCV is seen in a substantial number of chronic liver disease cases, who have a higher risk of progression to cirrhosis and decompensated liver disease and HCC [26], especially in the Asian-Pacific region, with the reported prevalence of HBV/ HCV infection at 10–16% [27]. 41 (4.1%) cases of HBV/HCV infection were included in our study: both EQ-5D index (0.785 ± 0.086 vs 0.771 ± 0.115, *p* = 0.791) and EQ-VAS (77.4 ± 14.7 vs 72.5 ± 16.0, *p* = 0.051) of patients without hepatitis B were higher than those of HBV/HCV patients. Cause no study has evaluated how HBV/HCV coinfection impact on the HRQoL except one focused on the impact of hepatitis B and C coinfection on health-related quality of life in HIV positive individuals [28], our results illuminated our understanding of this issue.

The impact of fatigue on quality of life of HCV-infected patients has long been a focused issue [29, 30]. However, the effect of other symptoms, such as malaise, appetite decrease, nausea, discomfort of right hypochondrium, on the HRQoL in patients with HCV has been overlooked. Questions were designed to assess uncomfortable symptoms, including malaise, appetite decrease, nausea, discomfort of right hypochondrium, fever, xanthochromia, xanthurenic and others, graded into mild, moderate and severe. Compared to no moderate or severe symptoms of discomfort, one or more symptoms were significant associated with worse EQ-5D index and EQ-VAS. Besides, more symptoms also brought more problems in dimensions of self-care, usual activity and pain/discomfort.

- As was well-known, impairment in HRQoL in patients with HCV was associated with the severity of liver disease, patients with decompensated cirrhosis, exhibiting the highest impairment in HRQoL [8, 31]. However, the number of subjects in end-stage liver disease (only 54 patients with decompensated cirrhosis) was small in 101 (10.1%) patients with cirrhosis of our study, compared with the vast majority of patients having HCV in the absence of cirrhosis. Nevertheless, cirrhosis were found to be significantly associated with lower EQ-5D index (β = − 0.114) and EQ-VAS (β = − 0.099) by impacting on all five dimensions. Very few former studies of HRQoL in HCV have been undertaken in patients with HCV-induced decompensated cirrhosis. That may be our concern in the future.

The limitation of this study was that it was a cross-sectional study without health population as control. Cause the patients we included were treatment naïve, we can’t tell the impact of antiviral therapy on HRQoL. We used a Japanese time trade-off value set to convert scores, from five dimensions (mobility; self-care; usual activities; pain/ discomfort; anxiety/depression), into a single index value for health status [13]. In spite of the widely usage of this Japanese time trade-off value set in Chinese population [32], it was still a limitation for our study. Future research is needed to validate the association between sociodemographic, clinical characteristics and HRQoL of patients in different liver disease stages of HCV. As the study of CCgenos is on-going, longitudinal assessment of HRQoL and influence of treatment on HRQoL will be performed.

## Conclusion

In this cross-sectional assessment of patients with chronic HCV infection in China, we have shown that HRQoL in Chinese HCV patients impaired. Significant association between HRQoL and sociodemographic and clinical factors such as residence, income, moderate or severe symptoms of discomfort, disease profile, hyperlipidemia and depression. However, further study of these association is needed. We have to focus on optimally managing care of patients with chronic HCV infection and improving their HRQoL.

## Abbreviations

ALD: Alcoholic liver disease
EQ-VAS: EQ visual analogue scale
HCC: Hepatocellular carcinoma
HCV: Hepatitis C virus
HRQoL: Health-related quality of life
NAFLD: Non-alcoholic fatty liver disease
